# An Analysis of the Food and Drug Administration Manufacturer and User Facility Device Experience Database for MAGnetic Expansion Control Spinal Rods

**DOI:** 10.1007/s43441-024-00724-4

**Published:** 2024-11-13

**Authors:** Jack Filan, Andrew Bowey, Thomas Joyce

**Affiliations:** 1 School of Medical Education, Newcastle University, Newcastle upon Tyne, NE1 7RU UK; 2 Great North Children’s Hospital, Royal Victoria Infirmary, Newcastle upon Tyne, UK; 3 School of Engineering, Newcastle University, Newcastle upon Tyne, NE1 7RU UK

**Keywords:** Early onset scoliosis, Magnetically controlled growing rods, MAGEC, MAGEC X, MAUDE, FDA

## Abstract

**Background:**

MAGnetic Expansion Control (MAGEC) rods can prevent repeated lengthening operations for scoliosis patients. However, there have been several Field Safety Notices issued, including a worldwide product recall due to actuator endcap separation. We aimed to review adverse events reported to the Food and Drug Administration (FDA) regarding MAGEC rods, focusing on MAGEC X.

**Methods:**

Reports submitted to the Manufacturer and User Facility Device Experience database in relation to MAGEC devices were accessed and analysed using R Statistical Software. Exclusion criteria included duplicate and literature review reports (*n* = 54). Free-text data were analysed using inductive content analysis.

**Results:**

1016 adverse events were reported to 11/30/2023. 99.0% (1006) were submitted by the manufacturer. Reports primarily arose from the UK (465, 45.8%) or US (421, 41.4%). From free-text data the most frequent adverse events were distraction mechanism failure (573), device wear (272), and actuator seal damage (180). Rod fracture (*n* = 48) was not significantly associated with rod diameter (≤ 5.0 mm or > 5.0 mm), *p* = 0.736. 234 reports referenced MAGEC X devices; actuator endcap separation was identified in 41.9% (99). Other events include failure of distraction (63), surface damage (31), and rod fracture (15). On 06/30/2020 MAGEC X2 received FDA approval. Twenty reports reference devices manufactured after this date, seven describe distraction mechanism failure; notably there are no reports of endcap separation.

**Conclusion:**

These data represent the largest series of adverse events reported for MAGEC rods, including significant new data regarding MAGEC X. As well as endcap separation, failure of distraction, surface damage, and rod fracture were reported.

## Introduction

Magnetically Controlled Growing Rods (MCGRs) are distraction-based systems used clinically in the management of Early Onset Scoliosis (EOS); defined as “scoliosis with onset less than 10 years of age, regardless of aetiology” [1–4]. MCGRs are lengthened using an external remote controller, which aims to prevent repeated surgery for rod lengthening [2, 5, 6].

The only commercially available MCGR system is the MAGnetic Expansion Control (MAGEC) rod, manufactured by NuVasive (NuVasive Specialised Orthopaedics, San Diego, CA) [2–4, 7]. MAGEC rods were licensed in Europe in 2009, and the US in 2014 [8, 9]. Since 2009 seven design iterations are known to have been commercially available [3].

**Fig. 1:**
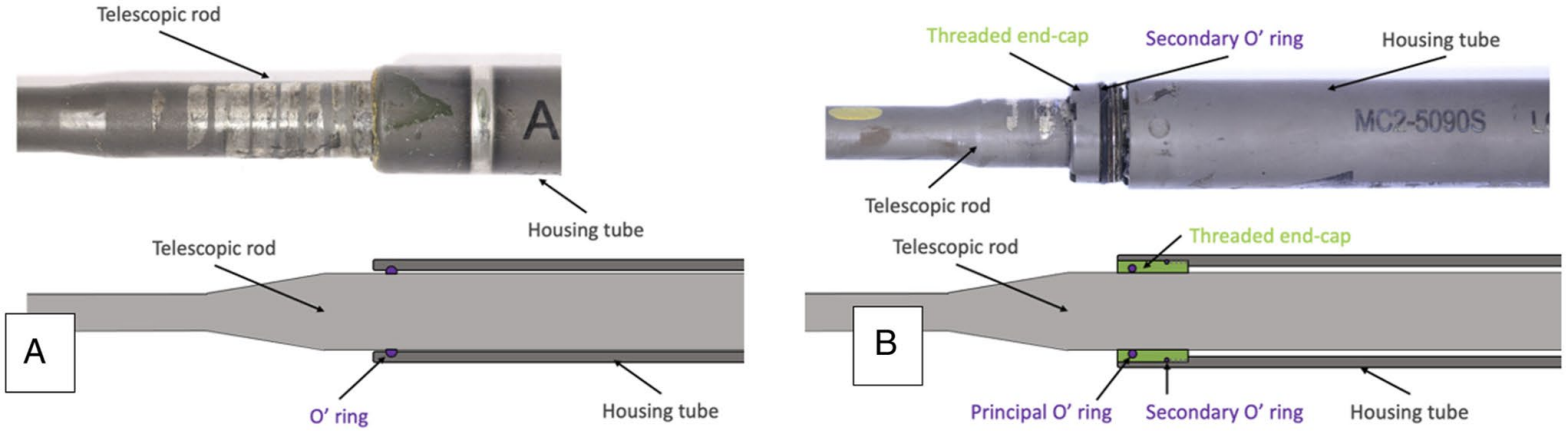
Photograph (top) and schematic (bottom) of changes to the actuator seal between MAGEC 1.3 (Panel A) and MAGEC X (Panel B) devices. Image source: Tognini et al., 2022.^3^ Reproduced under Creative Commons 4.0 without modification

The latest version, MAGEC X, has been commercially available since 2018 [10–13]. Previous devices used a single internal O-ring, as shown in Fig. 1a, to isolate the actuator from the surrounding environment, whereas MAGEC X introduced a threaded endcap component with two internal O-rings, as shown in Fig. 1b [3].

Safety concerns have been raised regarding MAGEC X rods. A Field Safety Notice (FSN) published on 13 February 2020 reported “post-implantation separation of an actuator endcap” [11], and instigated a global recall. A Medical Device Alert [14] and accompanying FSN [15] were published by the Medicines and Healthcare products Regulatory Agency (MHRA), the UK medical device regulator, on 1 April 2020 [14, 15]. These documents stated that no MAGEC devices of any model could be implanted in the UK (unless in exceptional circumstances) until further notice [14–16]. Endcap separation may be observed radiologically during implantation [3]. Previously reported failures of the O-ring seal are only identifiable following retrieval analysis [17].

On 25 March 2021 the CE (European Conformity) mark was temporarily suspended following an investigation by the notified body DQS Medizinprodukte GmbH [18, 19]. CE marking is required for all medical devices used in the European Union (EU), and confirms the device meets EU regulations [20]. The CE mark was later reinstated on 19 November 2021 with updated Instructions for Use (IFU) [21]. Additionally, an updated version of MAGEC X, known as MAGEC X2, was approved by the FDA in June 2020 and subsequently cleared for distribution in July 2021 [22–24]. In the UK, the MAGEC X system remained unavailable [25, 26] until the MHRA announced on 12 March 2024 that MAGEC X2 system may be used in the UK [27].

We aimed to investigate adverse events in relation to MAGEC rods reported to the US Food and Drug Administration (FDA) Manufacturer and User Facility Device Experience (MAUDE) database [28–31]. MAUDE contains reports of adverse events involving medical devices, including device malfunctions, serious injuries, and deaths [28–30]. MAUDE has been widely used to summarise adverse events of medical devices used in orthopaedics [32–43], and other specialities [44–55]. Previously Agarwal et al. [43] reviewed the available data for MAGEC rods to June 2019. However, this publication provides only superficial analysis of the available data. Furthermore, significant developments have occurred since this time, including the development of the MAGEC X and MAGEC X2 devices. We therefore aim to expand upon this publication and provide new topical information regarding the most contemporary MAGEC devices.

## Materials and Methods

Data used in this study were accessed from the MAUDE database via the FDA website [29, 31]. Reports submitted between 01/01/2014 and 12/31/2022 were accessed via downloadable data files [29]. Reports submitted from 01/01/2023 to 11/30/2023 were accessed via the MAUDE advanced search engine as the relevant data files are only updated on an annual basis [31]. The MAUDE search engine only allows a limited dataset to be downloaded [28], therefore records accessed via the search engine were completed by manual data recording. Exclusion criteria included duplicate reports, reports with insufficient information for analysis, and reports from published literature or literature review. Reports from published literature or literature review were excluded as citations or search term criteria were not provided within the reports to confirm the accuracy of the data.

The MAUDE dataset is divided across four primary and two supplemental files [28, 29]. The *DEVICE* file was first searched for “MAGEC”. Then the *MDR_REPORT_KEY* from each report was used to identify the corresponding record in all other files. Records in the *FREE_TEXT*, *DEVICE*, or *PATIENT_PROBLEM* files linked by the same *MDR_REPORT_KEY* were combined into a single record. Finally, all files were joined into a single dataset.

Data processing and analysis were performed using R Statistical Software (version 4.2.1 “Funny-Looking-Kid”), accessed via R Studio (“Cherry Blossom” Release (6e31ffc3 2023-05-09) for Windows) [56, 57]. Unless otherwise specified, all figures were produced using R Statistical Software.

Data analysis focused on descriptive statistics. Statistical tests, such as Chi-Squared test, were applied to investigate whether rod fracture was more frequent in smaller diameter devices and whether drive pin fracture was more frequent in earlier device versions. Rod diameters were classified as ≤ 5.0 mm or > 5.0 mm as described by previous authors [58]. Due to limited information, it was not possible to adjust for potential confounders including duration of implantation or patient age.

Relevant information, namely the device lot number, model number, and date of manufacture, were used to identify relevant device parameters. The lot number was used to identify the device diameter, length, and configuration, according to information publicly available from the manufacturer [59, 60]. Although most rods are implanted as a dual-rod construct, only a minority of reports provided these data for both devices.

Three device versions were denoted, summarised in Table 1. Device date of manufacture, where missing, was inferred from the device lot number. For MAGEC 1 and 2, example lot number A130227-01, characters 2–7 represent the date of manufacture as YY/MM/DD. Characters 8 and 9 represent the lot number from that day [17]. For MAGEC X, example 8062920AAA, characters 1–5 represent the date of manufacture as Y/MM/DD, in this example the date of manufacture is 06/29/2018. The significance of characters 6–10 is unknown. This approach has been validated using previous literature [17] and reports where both the lot number and date of manufacture were provided.

**Table 1:** MAGEC system device versions denoted and key identifying features of each version. Model numbers specified apply to US devices only

Device version	Date of manufacture	Model number	Lot number	Key design features
MAGEC 1	2009 to 27 July 2017	MS1___	A_____	Original device version and modifications thereafter
MAGEC 2	May 2015 to 27 July 2017	MC2___	A_____	5.0 mm and 6.0 mm rod diameter devices
MAGEC X	After 27 July 2017	MC2___	___AAA	Revised design with actuator endcap component

Descriptions of mechanical failure were explored via two techniques. Data on qualitative codes defined within the MAUDE database, namely ‘Device Problem Codes’, are reported. ‘Device Problem Codes’ are defined descriptions of device failure provided by the FDA [29]. One or more codes is attributed to each report by the reporter. Additionally, free-text data were analysed by the authors using inductive content analysis in Microsoft Excel (Version 2308 Build 16.0.16731.20052). Data coding was performed manually by a single author (JF), with regular data checking and discussion among all authors. Compared to a generic framework, such as the ‘Device Problem Codes’ used in MAUDE, inductive content analysis provides more detailed insights specific to this device. Codes were assimilated into broader themes, though these were synonymous in several cases.

Institutional ethical review was not required as the data studied is publicly available and there is no data which identifies individual patients.

## Results

Of 1072 adverse event reports identified, 56 were excluded (duplicate reports (*n* = 5), insufficient information (*n* = 3), and reports from published literature (*n* = 48)), leaving 1016 reports for further analysis.

The majority of reports arose from the UK (465, 45.8%) or US (421, 41.4%). Twenty-one (2.1%) events did not report the country source. 99.0% (1006) of reports were submitted by the manufacturer. The remaining ten were ‘voluntary reports’ by patients or caregivers, or ‘user facility’ reports submitted by healthcare facilities.

**Fig. 2:**
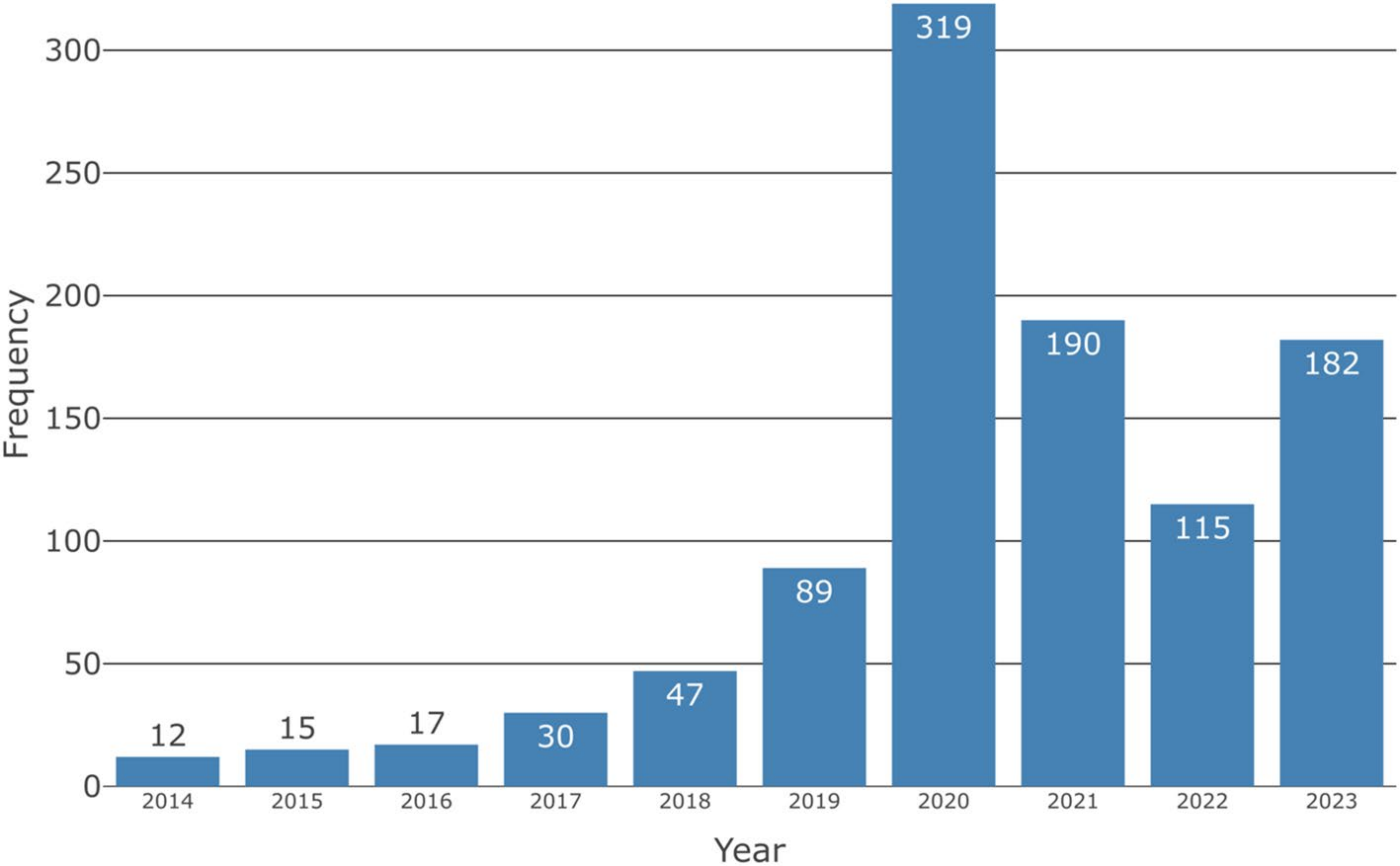
Bar chart representing the number of reports submitted per year. No missing data. 2023 data to 11/30/2023

Figure 2 represents the number of reports submitted per year. This peaks in 2020 at 319 reports. April 2020 is a notable outlier with 132 reports submitted this month. Whether this occurred in relation to COVID-19, FSNs published in February and April 2020 [11, 15], or another reason is unknown. Most records did not report the “date of event”, therefore reporting the time between an event occurring and this event being reported was not possible.

### Device Parameters

**Fig. 3:**
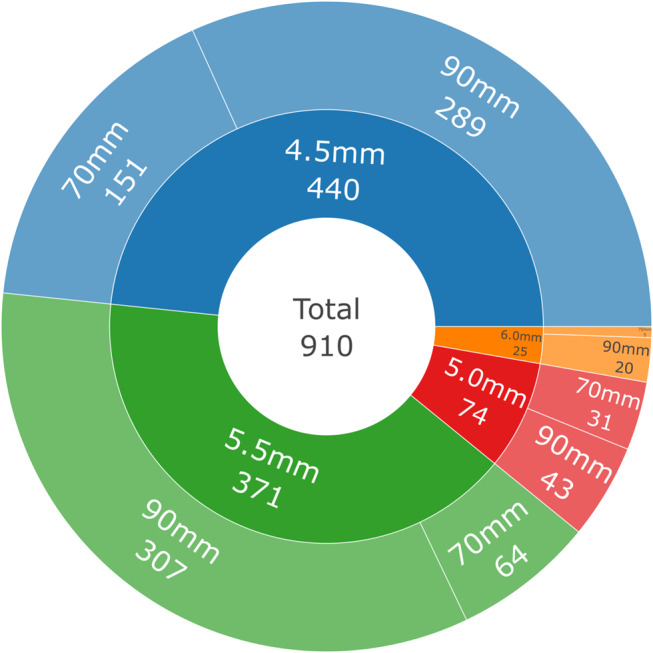
Sunburst plot of MAGEC rod diameter (inner ring) and actuator length (outer ring). Values represent the number of reports in each category, with data visualised hierarchically. Unknown or missing values excluded

910 model numbers were available for analysis; 36 reports provided two complete values, whilst the model number was not provided or incomplete in 142 reports. According to publicly available data, the model number was used to identify the actuator length and diameter of each device. These data are represented in Fig. 3.

904 lot numbers were available for analysis; 51 reports provided two lot numbers, the lot number was not provided or incomplete in 163 reports. Five lot numbers did not correlate to a valid date. In total the date of manufacture was identified for 851 reports. Table 2 represents the number of devices manufactured in each year in our dataset.

**Table 2:** Number of reported devices manufactured per year. Missing data included

Year of manufacture	Frequency
2010	5
2011	16
2012	44
2013	65
2014	173
2015	157
2016	117
2017	148
2018	84
2019	57
2020	27
2021	4
2022	4
2023	1
*Unknown*	*165*

Regarding device version, reports included 694 MAGEC 1 devices, 236 MAGEC X, and 20 MAGEC 2. Sixteen devices remained unclassified due to conflicting information between the lot and model numbers, likely related to data entry error. One hundred and four reports contained insufficient information to identify the device version. Of these data 54 devices were described as a second device within an existing report.

### Device Mechanical Failure

**Fig. 4:**
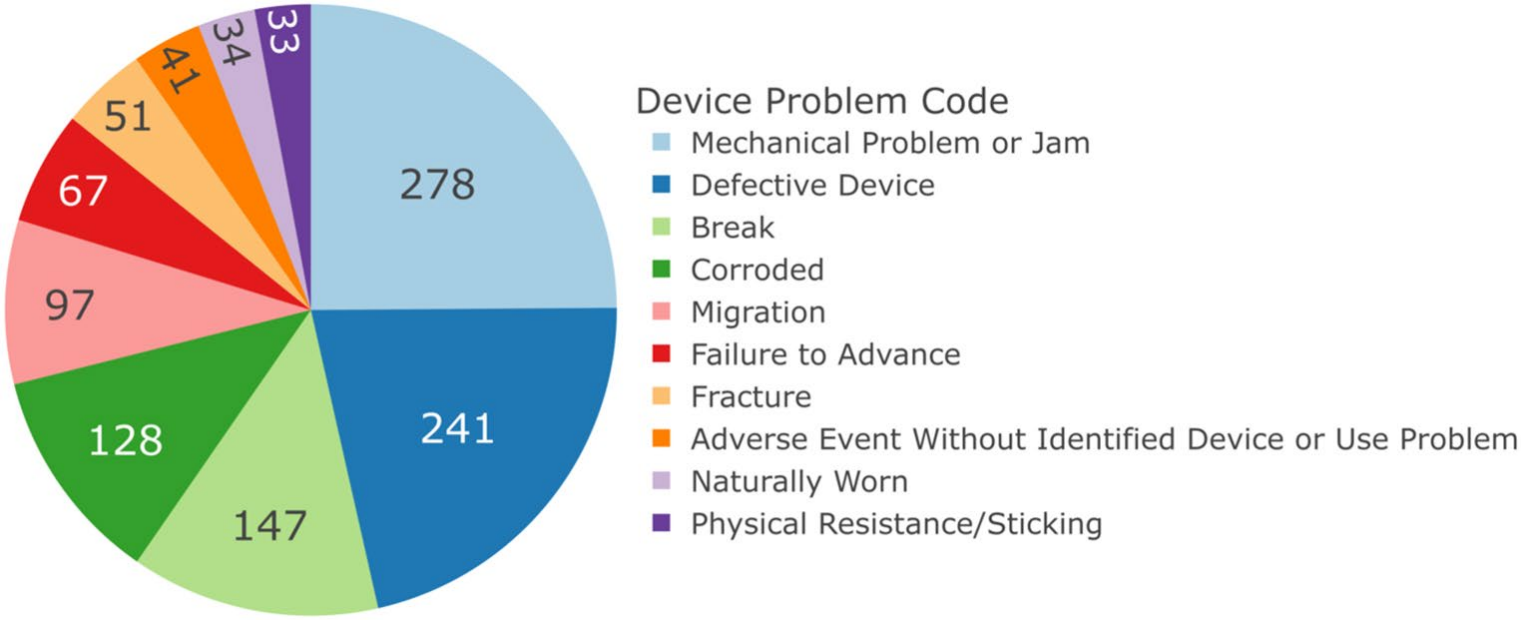
Pie chart representing frequency of Device Problem Codes, total 1393 codes. Codes utilising similar terminology combined. Codes with less than 30 events excluded

Total 1393 Device Problem Codes were provided for 1016 reports. Codes utilising similar terminology were combined, with the most frequent codes presented in Fig. 4.

**Fig. 5:**
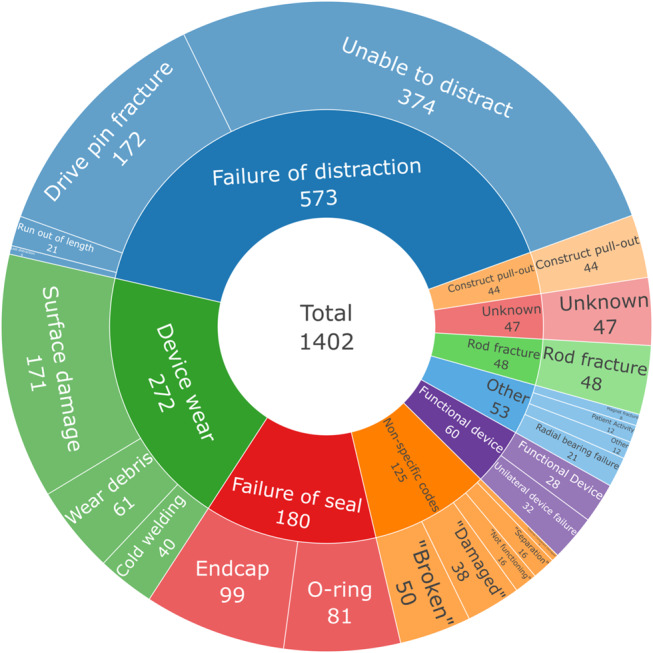
Sunburst plot of mechanical failure mechanism described by inductive content analysis. Inner ring describes major themes, with subcodes represented in the outer ring. No missing data

Total 1402 free-text codes were used for 1006 reports (Fig. 5). Failure of the distraction mechanism was the most frequently reported event, occurring in 573 (56.4%) reports. In 374 (36.8%) reports the rod was unable to lengthen, with 172 (16.9%) reporting drive pin fracture. Only 21 (2.1%) reports described the device reaching its maximum distraction length.

Device wear or cold welding was frequently reported, either as surface damage (171), wear debris within the actuator (61), or cold welding (40). These findings were only reported in devices that underwent retrieval analysis, which includes 475 (46.8%) reports. Failure of the seal encompasses endcap separation (99, 9.7%) and O-ring damage (81, 8.0%).

Non-specific language was used in 125 (12.3%) reports, which limited further analysis. Terms included “broken” (50), “damaged” (38), “not functioning” (16), “separation” (16), and “internal component damage” (5). Whilst these may appear synonymous with other reports, significant ambiguity remains, for example “broken” may describe rod fracture or failure to lengthen, therefore these descriptors were coded separately.

Forty-eight rod fracture events and 44 construct pull-outs were detected. Infrequent but notable mechanisms of failure included damage to the radial bearing (21) and magnet fracture (8).

In 28 reports the device was reported as being functional despite having been explanted. In 32 reports exactly one device in dual-rod construct remained functional. Finally, the mechanism of failure was unknown in 47 reports.

### MAGEC X

We identified 236 MAGEC X devices across 234 reports. This value is likely an underestimate considering the device version was unknown in 104 reports. 150/234 reports listed lot numbers included in the February 2020 MAGEC X recall [11]. Of the 84 MAGEC X reports not identified in this recall, many of these reports did not provide a lot number, which is the only device identifier within the recall notice.

Endcap separation was the most frequent mechanism of mechanical failure, occurring in 41.9% (99) of MAGEC X reports. However, other events continue to occur including failure of distraction (63, 26.9%), surface damage (31, 13.2%), and rod fracture (15, 6.4%).

Within the MAUDE data, we identified that the first report of endcap separation was submitted in November 2018 [MDR_REPORT_KEY 8040420]; eleven further reports were submitted prior to the FSN publication on 13 February 2020 [11]. This FSN reports endcap separation had occurred in 0.5% of devices [11, 61], the methodology for this figure has not been reported. The FDA additionally report 3502 devices were recalled [11, 61], which provides an event rate of 0.34% when eleven events have occurred.

Regarding MAGEC X2, 20 MAGEC X reports list devices manufactured after 06/30/2020, the date on which MAGEC X2 was approved by the FDA [22]. We therefore infer these devices to represent MAGEC X2 versions. Seven reports describe failure of the distraction mechanism; notably there are no reports of actuator endcap separation in this version to 11/30/23.

### Rod Fracture

Forty-eight rod fractures were reported, of these 34 reported the rod diameter. Regarding device diameter, 489 (48.1%) reports listed devices with diameter ≤ 5.0 mm, 384 (37.9%) were > 5.0 mm, diameter was unknown in 143 (14.0%) reports (Table 3). Rod diameter was categorised as ≤ 5.0 mm or > 5.0 mm to provide consistency with previous studies [58]. No significant difference in rod fracture was observed according to rod diameter in the available data, X^2^, 1, *n* = 873, 0.113, *p* = 0.736.

**Table 3:** Incidence of rod fracture as stratified by rod diameter. Reports with an unknown rod diameter (*n* = 132) excluded

	No rod fracture	Rod fracture	Percentage fractured	Total
Rod diameter ≤ 5.0 mm	469	20	4.3%	489
Rod diameter > 5.0 mm	370	14	3.8%	384
Total	839	34	4.1%	873

### Drive Pin Fracture

As of 26 March 2015, modifications were made to strengthen the drive pin [62]. We identified 172 reports of drive pin fracture, of these 163 reported the manufacture date (Table 4). There is a significantly increased risk of drive pin fracture in reports of devices manufactured before 26 March 2015 (row 1) compared to reports listing devices manufactured after this date (row 2), X^2^, 1, *n* = 663, 48.654, *p* < 0.001.

**Table 4:** Incidence of drive pin fracture stratified by device date of manufacture (before or after 26 March 2015) and MAGEC X device. Reports with an unknown date of manufacture or device version (*n* = 120) were excluded

	No drive pin fracture	Drive pin fracture	Drive pin fracture rate (%)	Total
Manufactured before 26 March 2015	193	109	56.5	302
Manufactured after 26 March 2015	314	47	15.0	361
MAGEC X	226	7	3.1	233
Total	733	163	N/A	896

In addition, MAGEC X has a “reinforced locking [drive] pin” [12]. We therefore compared drive pin fracture events in MAGEC X (row 3) reports to reports of devices manufactured after 26 March 2015 (row 2). Within this dataset we identified a significantly reduced risk of drive pin fracture in MAGEC X compared to rods manufactured after 26 March 2015, X^2^, 1, *n* = 594, 17.186, *p* < 0.001.

## Discussion

This study aimed to evaluate the adverse event profile of MAGEC rods using the MAUDE database. These data represent by far the largest series of adverse event reports available for the MAGEC system, totalling 1016 reports. These data are particularly important for the MAGEC X and MAGEC X2 systems, which remain in clinical practice in Europe [21, 63] and the US [23, 24], but have a limited literature base to date [3].

### MAGEC X

To date, only one published study is available specifically regarding MAGEC X [3]. This paper describes a comparative retrieval analysis between MAGEC X and MAGEC 1.3 devices, conducted by the London Implant Retrieval Centre (LIRC) [3]. Fifteen rods from ten patients were included per group. Overall, similar performance results were observed in both groups [3]. This study also experimentally assessed the torque required to loosen the endcap, reporting a median peak loosening torque of 24.9 LBFin [3]. At the time of publication, there was no established standard for peak loosening torque.

**Fig. 6:**
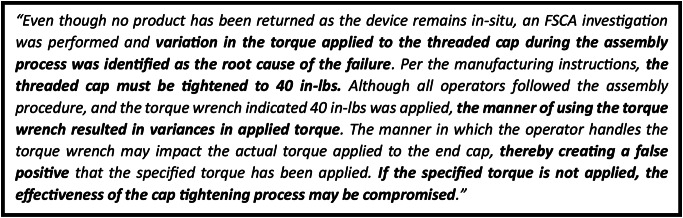
Notable manufacturer narrative repeated verbatim in at least ten reports of actuator endcap separation. Quoted from report number 11092282. Key statements highlighted in bold. FSCA – field safety corrective action

Within MAUDE we have identified new information, Fig. 6, to contextualize this result. According to the manufacturer, the threaded endcap should be tightened to 40 LBFin during assembly. 24.9 LBFin would appear a significant deviation from this value. Whether this deviation occurs from differing measurement techniques, calculation of the median, or another reason is unclear.

### Drive Pin Fracture

Drive pin fracture is a significant event as the device is unlikely to continue lengthening, and may ‘piston’ during compressive loading which can generate titanium debris [62]. In addition to the local effect of titanium debris, metallosis, recent evidence shows the majority of patients implanted with MCGRs have elevated serum metal ion levels [64–66]. Devices manufactured after 26 March 2015 were modified with the drive pin manufactured from a stronger material and in a larger diameter [67]. In addition, MAGEC X reportedly has a “reinforced locking [drive] pin” [12].

Our data confirm drive pin fracture is significantly more frequent in devices manufactured before 26 March 2015 (56.5%) than later versions (15.0%), *p* < 0.001. Similar event rates have been reported in explant studies performed by Newcastle University [67] (Joyce et al.) and LIRC [7] (Tognini et al.) (Table 5). These centres, alongside MAUDE, typically receive ‘failed’ devices, therefore a greater event rate may be expected. However, it remains concerning that the device manufacturer reported a 5% and 0% risk respectively [62].

**Table 5:** Comparison of drive pin fracture rates reported by two published studies and a Field Safety Notice published by the manufacturer [7, 62, 67]. Sample size refers to the number of devices. Both studies assessed drive pin fracture during device disassembly

Source	Sample size	Before 26 march 2015	After 26 march 2015
Joyce et al. 2020	134	59%	21%
Tognini et al. 2023	76	52%	18%
Field Safety Notice 2019	Unknown	5%	0%

Additionally, our data confirm drive pin fracture is significantly less frequent in MAGEC X rods than devices manufactured after 26 March 2015, X^2^*p* < 0.001. This is the first available data regarding drive pin fracture in MAGEC X. This is a positive finding, though clinicians should remain vigilant that drive pin fracture can occur in MAGEC X devices.

### Limitations

The MAUDE database has several limitations which should be considered when interpreting the results of this work. MAUDE is a voluntary reporting system, therefore allowing the possibility of missing data [32, 68], inaccurate, or unverified reports. A voluntary reporting system is also prone to under-reporting, and no further information is available regarding the frequency of device use to quantify the degree of under-reporting. Finally, the majority of reports are submitted by device manufacturers, rather than healthcare professionals [28, 32, 69]. This creates difficulty in extracting meaningful clinical information.

The other key issue with MAUDE and its analysis is, what is the denominator? In our case, how many children have had MAGEC rods implanted? This information is not available from the manufacturer. This is a significant challenge for all authors presenting pharmacovigilance analyses across a range of medical devices. However, data from the LIRC published in May 2019 stated that 3,000 patients worldwide had received MAGEC rods [70]. We estimate that a further 3,000 children could have received these implants from May 2019 to the end of our data collection period (11/31/2023). Based on this estimate, the 1016 adverse events we report represent about 1 in 6 of the children implanted with MAGEC rods. Most children have two MAGEC rods implanted as a dual-rod construct, therefore our estimate of 6,000 children would equate to 12,000 devices implanted. Given that 3502 MAGEC X rods were recalled in February 2020 [61], 12,000 seems plausible. We would be overreaching to speculate on the number of MAGEC X rods compared with earlier iterations, given the worldwide recall, as well as the possibility of increasing numbers implanted over time as the MAGEC device became established. For this latter reason, we also hesitate to estimate the number of MAGEC rods implanted per year.

Specific to this device, several data fields, notably device duration of implantation, were not included within the suggested dataset and therefore rarely included within reports. In addition, a lack of unique device identifiers presented challenges in identifying duplicate reports. Within MAUDE, information on any second device was variably reported; although MAUDE provides a mechanism to link reports this was not utilised when reporting the data. Therefore, we were unable to confirm the exclusion of duplicate reports, especially where two devices from a dual-rod construct *in the same patient* have been submitted separately.

Despite these limitations, there remains interest in the MAUDE data for MAGEC rods as they represent the largest series of adverse event reports available for this device to data and can provide valuable insight into the adverse event profile of this device. Within this study we have accessed the complete MAUDE dataset, rather than the limited version available from the MAUDE search engine [28, 32]. This provides a more complete picture than other studies, notably including Agarwal et al., [43] where the complete dataset was not accessed.

## Conclusions

These data represent largest series of adverse events reported for MAGEC rods to date. MAGEC rods remain in clinical use in the US and Europe for patients with Early Onset Scoliosis. We recommend continued vigilance and shared decision-making with patients and their families among clinicians using MAGEC X or MAGEC X2 devices. Further comparative studies are necessary to consider complications and other endpoints such as unplanned return to theatre, alongside clinical efficacy. These questions may also be answered by an open-access clinical registry, providing sufficient patient and device specific data was collected.

Endcap separation is the most frequent mechanism of failure reported for MAGEC X, occurring in 44% of reports. However, other mechanisms of failure continue to affect the MAGEC X system. Therefore, we cannot conclude that the design modifications taken have resolved these fundamental challenges. Rod diameter did not affect the occurrence of rod fracture events. We confirm that drive pin fracture was significantly more common in devices manufactured before 26 March 2015. Our data also indicates a reduced risk of drive pin fracture in MAGEC X devices.

## Data Availability

Data used in this study is publicly available via the United States Food and Drug Administration, URL: https://www.accessdata.fda.gov/scripts/cdrh/cfdocs/cfmaude/search.cfm.
